# A Study on the Diagnostic Value of Dual‐Energy CT (DECT) Imaging in Patients With Gouty Arthritis

**DOI:** 10.1111/1756-185X.15431

**Published:** 2024-12-01

**Authors:** YiXin Luan, XingShuai Gao

**Affiliations:** ^1^ Department of Medical Iconography Zhongshan Orthopaedic Hospital Zhongshan Guangdong China; ^2^ Department of Orthopaedic Surgery Zhongshan Orthopaedic Hospital Zhongshan Guangdong China

**Keywords:** diagnostic value, dual‐energy computed tomography, gouty arthritis, X‐ray

## Abstract

**Objectives:**

To explore the diagnostic value of dual‐energy computed tomography (DECT) in patients with gouty arthritis.

**Methods:**

A total of 160 patients with gouty arthritis who were treated in our hospital from January 2023 to October 2023 were selected as the research subjects. The participants were randomly divided into two groups: an observation group and a control group. Each group had 80 cases. Observation group performed DECT examination and control group performed X‐ray examination. The researchers recorded the general information of the participants in both groups and conducted single‐factor analysis. They compared the detection of positive diseased joints between the two groups, as well as compared the distribution of positive diseased joints. In addition, the number of joint lesions was compared in the two groups.

**Results:**

There were not any statistically great differences between the two groups in gender, age, BMI, disease duration, blood uric acid, and erythrocyte sedimentation rate (*p* > 0.05). During the DECT examination, a total of 82 positive diseased joints were identified in the observation group. The observation group also had a positive rate of 85.42% (82/96) in the DECT examination. While the control group had a total of 55 positive diseased joints during the X‐ray examination with a positive rate of 59.78% (55/92). The difference between the two groups of patients in the positive rate of two kinds of examination was statistically significant (*χ* ^2^ = 15.616, *p* < 0.001). No statistical significance was found in the distribution of the number of positive diseased joints between two groups (*χ* ^2^ = 1.986, *p* = 0.851). Both DECT and X‐ray examinations of patients in the two groups revealed that the lesions were primarily located in the soft tissues or ligaments surrounding the distal small joints of the limbs, such as metatarsophalangeal joints, ankle joints, and proximal interphalangeal joints. Compared with the X‐ray examination of the patients, the DECT examination of the patients showed a great increase in the number of bone destruction, gout nodules, and soft tissue swelling (*χ* ^2^ = 7.712, 10.441, 5.389, *p* = 0.005, 0.001, 0.020). Moreover, the DECT examination of patients showed the presence of urate crystals and joint effusion, while the X‐ray examination of patients in the control group did not show any.

**Conclusions:**

Siemens dual‐source 64‐slice CT dual‐energy imaging has better diagnostic value for gouty arthritis than X‐ray and has higher specificity for detecting urate crystals and joint effusion. Therefore, Siemens dual‐source 64‐slice CT dual‐energy imaging examination may serve as a promising new noninvasive technique for early diagnosis, clinical screening, and follow‐up of gout in the future.

Gout arthritis is a crystal‐related arthropathy caused by urate deposition and is considered both, an inflammatory and metabolic disease [1]. It is more common in middle‐aged and elder men with age over 40. Hyperuricemia is closely related to the occurrence and development of gouty arthritis. In cases of gout, the body's uric acid metabolism is disrupted, resulting in an increase in uric acid levels, a condition known as hyperuricemia. Over time, urate crystal deposition occurs in the patient's joints, cartilage, synovium, and other parts, triggering the inflammatory reaction that eventually triggers chronic gouty arthritis [2]. In addition, genetic factors, dietary habits, chronic kidney diseases, joint injuries, obesity, and so on may contribute cause [3].

In the acute stage of gout, there is typically severe joint swelling and pain. However, these symptoms are self‐limiting, meaning they can resolve spontaneously within a few days or 2 weeks [4, 5]. In the acute stage, the joints are red, swollen, hot, and painful, accompanied by general weakness, headache, and fever. In the chronic phase, related complications such as gouty nephritis, cerebrovascular disease, and tophi occur. With the recurrence of gouty arthritis, the condition tends to worsen over time. The acute phase attacks become prolonged and the intermittent periods become shorter. The joint damage gradually worsens, and joint deformity and stiffness may occur in the late stage. As the disease progresses, crystal deposition continues in the joints and soft tissue, manifesting as a collection of crystals, inflammatory cells, and fibrous tissue. Collectively known as tophi, the deposits cause gouty bone destruction, joint deformity, and disability [6]. For patients with gouty arthritis, early diagnosis, intervention, and treatment are particularly important. Examination of synovial fluid is indeed considered the “gold standard” for diagnosing gouty arthritis. Polarized light microscopy is used to check whether there are urate crystals in synovial fluid. However, it does come with certain limitations that limit synovial fluid to be widely used clinically, such as difficulties in implementation and easy infection [7]. Currently, the diagnostic criteria for gouty arthritis based on clinical characteristics established by the American College of Rheumatology are mostly used in clinical. However, in addition to clinical symptoms, the diagnosis still needs to consider other factors, including laboratory tests, imaging tests, and so on [8, 9]. At this stage, clinical diagnosis of chronic gouty arthritis is mostly done through imaging examinations such as ultrasound, X‐ray, computed tomography, and DECT. Although the methods mentioned above are commonly used, examining the contents of synovial fluid remains the “gold standard” for diagnosing gouty arthritis. Polarized light microscopy is used to examine the presence of urate crystals in synovial fluid. However, aspiration of already painful and swollen joints may cause additional trauma and put the patient at risk for infection. What is more, joint fluid may be excessively extracted during the examination, resulting in a decrease in intra‐articular pressure and causing further discomfort or pain [10, 11].

DECT is a new imaging detection method that is favored by diagnosis and treatment in clinical orthopedics, urology, and other fields. It is capable of replacing traditional invasive examination methods [12]. DECT technology can significantly improve the accuracy of lesion detection and classification. It can also accurately identify different substances, such as calcification and hemorrhage, which is crucial for the development of diagnosis and treatment plans [13]. This technology uses the difference in the attenuation degree of X‐ray beams of different energies after passing through the material to separate and image the components of the material, providing more accurate and reliable information for medical diagnosis and treatment. DECT technology can be used to detect and identify different components in substances, such as bone, soft tissue, bleeding, and so on, and has been widely used in spinal fractures, urinary stones, and so on [14, 15]. Additionally, DECT technology can also provide more physiological and pathological information to help doctors better understand the patient's condition and formulate more precise treatment plans. In clinical practice, DECT is used in both diagnosis and monitoring of many diseases. For example, in bone and joint diseases, DECT can be used to evaluate the shape and density of articular cartilage to diagnose and monitor diseases such as osteoarthritis and bone hyperplasia. In addition, DECT can also be used for the diagnosis of lung diseases, such as pulmonary nodules, lung cancer, and so on [16]. Therefore, this study aims to include patients with gouty arthritis as research subjects and discover the diagnostic value of DECT in patients with gouty arthritis.

## 1 Objects and Methods

### 1.1 Research Objects

A total of 160 patients with gouty arthritis who were treated in our hospital from January 2023 to October 2023 were selected. The participants were randomly divided into two groups: an observation group and a control group. Each group had 80 cases. Inclusion criteria: (1) Meet the diagnostic criteria for gouty arthritis recommended by the American College of Rheumatology [17]; (2) Patients are over 18 years old; (3) Patients have high compliance and accept relevant examinations; (4) Clinical data are full; (5) All patients have been given informed consent. Exclusion criteria: (1) Patients with bone‐related diseases, such as fractures, osteomyelitis, and so on; (2) Patients having a history of chronic gouty arthritis. Patients are receiving uric acid‐lowering treatment; (3) Patients with contraindications to relevant examinations; (4) Patients with mental illness who are unable to cooperate with the examination. This study has been reviewed and approved by the Ethics Committee of our hospital and obtained an ethics certificate. Based on the principle of confidentiality, we strictly keep the personal information and family information of patients with gouty arthritis confidential. Detection of needle‐shaped birefringent uric acid crystals in joint fluid or tophi aspirates through light microscopy is the “gold standard” for the diagnosis of gouty arthritis. All patients underwent this examination. After examination, it was found that there were 96 joint lesions in the observation and 92 joint lesions in the control.

## 2 Method

### 2.1 Inspection Method

Observation group: Use Siemens dual‐source 64‐slice CT scanner (SOMATOM Definition Flash syngo CT) for examination, and conduct dual‐energy scans on major joints throughout the body, such as bilateral knees, ankles, elbows, hands, feet, and so on. The affected anatomical area must be located in the center of the scanning area as much as possible. The appropriate dual‐energy imaging sequence for scanning with a CT value range of 150–500 Hu was selected. The urate deposition imaging and volume were obtained through postprocess and reconstruction. Transverse, coronal, and sagittal multiplane pseudocolor and volumetric rendering images were completed with green representing MSU crystals. Through postprocessing and reconstruction, the urate is obtained. Transverse, coronal, and sagittal multiplane pseudocolor images of the crystal and volumetric rendering (VR) pseudocolor images of urate crystals. The green images are salt crystals.

Control group: X‐rays were irradiated by using a Philips DR X‐ray machine. The patient took anteroposterior or oblique films of the diseased joints. The ankle joint was 60 kV, the foot was 57 kV, the knee joint was 66 kV, the hand was 52 kV, and the wrist and elbow joints were 60 kV.

### 2.2 Image Analysis

In this study, the DECT and X‐ray examination results were read by two experienced radiologists using a double‐blind method to record bone destruction, urate crystal deposition, gout nodule (tophi), soft tissue swelling, joint effusions, and so on In case of disagreement, a third, blinded radiologist was brought in to determine the final read.

### 2.3 Observation Indicators

(1) Record the general information of all patients: gender, age, BMI, disease duration, blood uric acid, and erythrocyte sedimentation rate. (2) Compare the detection of positive diseased joints between the two groups; (3) Compare the distribution of positive diseased joints between the two groups; (4) Compare the amount of joint lesions between the two groups.

### 2.4 Statistical Analysis

Measurement indicators that follow a normal distribution were recorded as mean ± standard deviation. The independent sample *t‐*test was utilized to compare between different groups. The statistical analysis was performed by using SPSS 21.0. Count indicators were recorded as [number of cases (percentage)]. The *χ* ^2^ test was employed to compare between groups. *p* < 0.05 was considered as a statistically significant difference.

## 3 Results

### 3.1 Comparison of the General Information of the Two Groups

No significant differences were observed between the two groups in terms of gender, age, BMI, disease duration, blood uric acid, and erythrocyte sedimentation rate (*p* > 0.05) (see Table 1).

**TABLE 1:** Comparison of the general information.

Index	Observation group (*n* = 80)	Control group (*n* = 80)	*χ* ^2^/t	*p*
Gender (*n*, %)			0.317	0.573
Male	62 (88.6)	64 (91.4)		
Female	8 (11.4)	6 (8.6)		
Age	46.88 ± 8.26	47.14 ± 8.19	0.202	0.840
BMI (kg/m^2^)	22.15 ± 0.80	22.23 ± 0.82	0.625	0.533
Duration of disease (years)	2.05 ± 0.55	2.08 ± 0.44	0.317	0.752
Serum uric acid (μmol/L)	455.66 ± 40.24	460.34 ± 43.29	0.708	0.480
Erythrocyte sedimentation rate (mm/h)	30.26 ± 5.25	31.29 ± 5.58	1.197	0.233

### 3.2 Comparison of the Detection of Positive Diseased Joints Between the Two Groups

**FIGURE 1:**
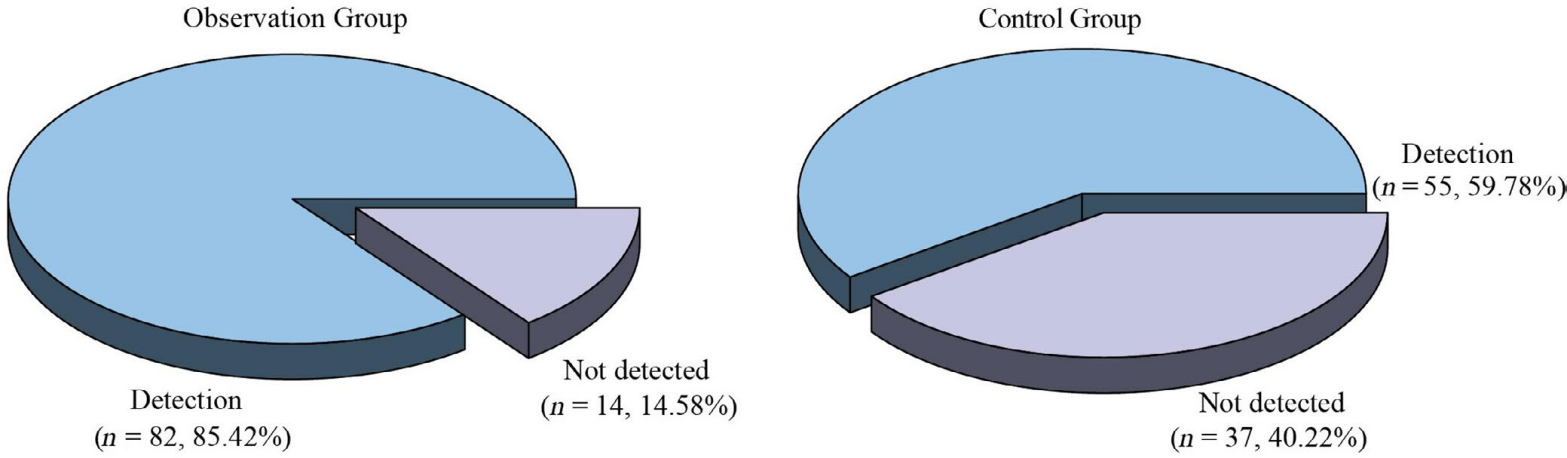
Three‐dimensional pie chart comparison of positive disease joint detection in two groups.

During the DECT examination, a total of 82 positive diseased joints with chronic gouty arthritis were identified in the observation group. The observation group also had a positive rate of 85.42% (82/96). While the control group had a total of 55 positive diseased joints during the X‐ray examination with a positive rate of 59.78% (55/92). The difference between the two groups in the positive rate of two kinds of examination was statistically significant (*χ* ^2^ = 15.616, *p* < 0.001) (see Figure 1).

### 3.3 Comparison of the Distribution of Positive Diseased Joints Between the Two Groups

There was no statistically great difference in the distribution of the number of positive diseased joints with chronic gouty arthritis between the observation group and the control group (*χ* ^2^ = 1.986, *p* = 0.851).

**FIGURE 2:**
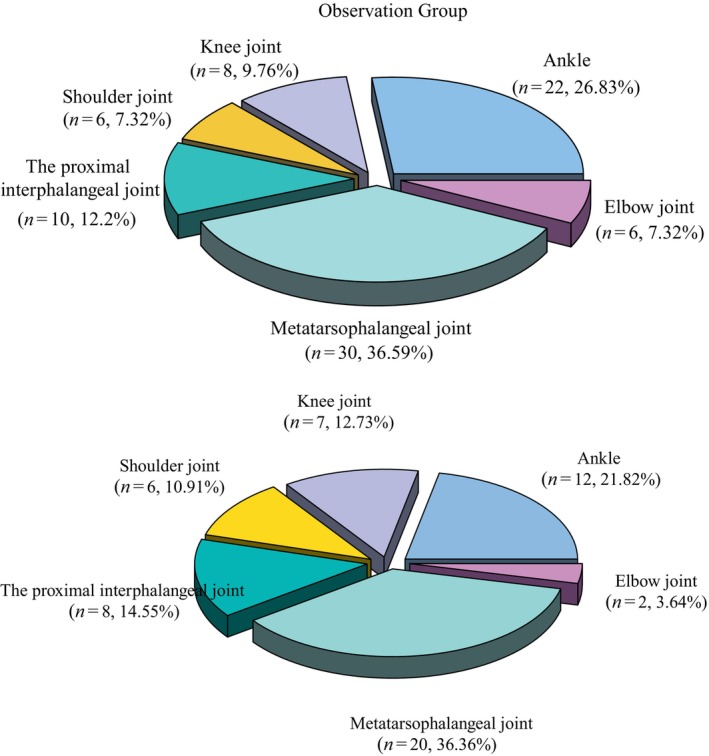
Three‐dimensional pie chart comparison of the distribution of positive diseased joints between the two groups.

Those patients who underwent DECT examination in the observation and patients who underwent X‐ray examination in the control group found that the lesions were mainly distributed in the soft tissues or ligaments surrounding the distal small joints of the limbs such as metatarsophalangeal joints, ankle joints, and proximal interphalangeal joints (see Table 2 and Figure 2).

**TABLE 2:** Comparison of the distribution of positive diseased joints between the two groups (*n*, %).

Group	Observation group (*n* = 80)	Control group (*n* = 80)	*χ* ^2^	*p*
Gold standard	96	92	—	—
Number of positive diseased joints	82	55	—	—
Ankle joint	22 (26.8)	12 (21.8)	1.986	0.851
Knee joint	8 (9.8)	7 (12.7)		
Shoulder joint	6 (7.3)	6 (10.9)		
Proximal interphalangeal joint	10 (12.2)	8 (14.6)		
Metatarsophalangeal joint	30 (36.6)	20 (36.4)		
Elbow joint	6 (7.3)	2 (3.6)		

### 3.4 Comparison of the Number of Joint Lesions Between the Two Groups

The patients who underwent DECT examination in the observation group presented a dramatic increase in the number of bone destruction, gout nodules, and soft tissue swelling compared to the patients who underwent X‐ray examination in the control group (*χ* ^2^ = 7.712, 10.441, 5.389, *p* = 0.005, 0.001, 0.020). Moreover, the patients who underwent DECT examination in the observation group showed urate crystals and joint effusion, while the patients who underwent X‐ray examination in the control group showed no urate crystals and hydrops articuli (see Table 3).

**TABLE 3:** Comparison of the number of joint lesions between the two groups (*n*, %).

Group	Observation group (*n* = 80)	Control group (*n* = 80)	*χ* ^2^	*p*
Gold standard	96	92		
Number of positive diseased joints	82	55		
Bone destruction	68 (70.83)	47 (51.09)	7.712	0.005
Gouty nodules	70 (72.92)	46 (50.00)	10.441	0.001
Soft tissue swelling	65 (67.71)	47 (51.09)	5.389	0.020
Urate crystals	136	0	—	—
Hydrops articuli	90	0	—	—

## 4 Discussion

Over the last few decades, the incidence of gout and acute gouty arthritis has increased for several reasons, including diet, lifestyle changes, and aging. This trend has gradually become a public health concern, necessitating the need for better modalities to diagnose (and manage) the disease [18]. Gouty arthritis typically manifests with sudden and acute episodes of joint inflammation. A total of 160 patients with gouty arthritis who were treated in our hospital were selected as the research subjects. The participants were randomly divided into two groups: an observation group and a control group. After the detection of needle‐shaped birefringent uric acid crystals in joint fluid or tophi aspirates through light microscopy, 96 joint lesions in the observation group and 92 joint lesions in the control group were found. During the DECT examination, a total of 82 positive diseased joints were identified in the observation group. The observation group also had a positive rate of 85.42% (82/96), while the control group had a total of 55 positive diseased joints during the X‐ray examination with a positive rate of 59.78% (55/92). The difference between the two groups in the positive rate of two kinds of examination was statistically significant. The results indicate that the detection rate of DECT examination is high and is similar to the results of previous studies [19]. Comparing the distribution of positive diseased joints between the two groups, it was found that there was no statistical significance in the distribution of the number of positive diseased joints. DECT and X‐ray examination of both the observation group and the control group revealed that the lesions were primarily localized in the soft tissues or ligaments surrounding the distal small joints of the limbs such as metatarsophalangeal joints, ankle joints, and proximal interphalangeal joints. These findings are consistent with previous results of studies [20].

X‐ray is an imaging method commonly used to detect gouty arthritis. It has many advantages such as fast, simple, noninvasive, and low cost [21, 22]. It can present images of joints immediately as needed. It can also be used in patients with early gouty arthritis. Soft tissue swelling can be seen in plain radiographs [23]. As the disease progresses, X‐ray films of advanced patients may reveal round or irregular‐shaped translucent areas resembling punch holes in the bone near the joint ends. These areas can also appear honeycomb‐shaped or cystic, with normal or increased density in the surrounding bone. The boundaries of these lesions are clear, and the joint surfaces may appear uneven. Additionally, there may be a narrowing of the joint spaces. All of these findings can help physician quickly understand the condition of the joints [24, 25]. In this study, X‐ray examination of the patients in the control group identified a total of 47 bone lesions, 46 gout nodules, and 47 soft tissue swellings. In addition to X‐ray examination, other imaging examination methods such as ultrasound, CT, and MRI can also be used for the diagnosis of gouty arthritis. These examination methods can provide more detailed images of joint structures and surrounding soft tissues, helping to diagnose gouty arthritis more accurately and provide a more comprehensive assessment of the condition. In our study, DECT examination of patients in the observation group revealed 68 bone destructions, 70 gout nodules, and 65 soft tissue swellings. The number of bone destructions, gouty nodules, and soft tissue swellings detected in the observation group increased significantly compared to the control group. The result suggested that DECT is superior to X‐ray in detecting bone destruction, gout nodules, and soft tissue swelling.

Dual‐energy X‐ray spectroscopy used by DECT can acquire two different data sets at different energies to improve the ability to detect lesions. In this study, DECT examination of patients in the observation group revealed the presence of 136 areas of crystal deposition and 90 joint effusions. Our study demonstrated that DECT can become a diagnostic tool of great importance for gouty arthritis, helping to diagnose gouty arthritis more accurately and provide a more comprehensive assessment of the condition. However, this study also has some shortcomings. Our study was a single‐center study with a small and single sample size, and a larger sample size is needed to verify our results in the future.

In summary, Siemens dual‐source 64‐slice CT dual‐energy imaging has better diagnostic value for gouty arthritis than X‐ray. It also has higher specificity for urate crystals and joint effusion. Therefore, Siemens dual‐source 64‐slice CT dual‐energy imaging examination may be used as a new and noninvasive technical means for early diagnosis, clinical screening, and review of gout in the future.

## Author Contributions

YiXin Luan and XingShuai Gao carried out study concepts and design, literature research, clinical studies, and manuscript preparation; YiXin Luan contributed to data acquisition and analysis; XingShuai Gao helped with manuscript editing and review.

## Ethics Statement

All procedures performed in studies involving human participants were in accordance with the 1964 Helsinki Declaration and its later amendments or comparable ethical standards. This study is approved by the Ethics Committee of Zhongshan Orthopedic Hospital. Written informed consent was obtained.

## Consent

Informed consent was obtained from all individual participants included in the study.

## Conflicts of Interest

The authors declare no conflicts of interest.

## Data Availability

The data that support the findings of this study are available from the corresponding author upon reasonable request.
